# Multi-decadal warming alters predator’s effect on prey community composition

**DOI:** 10.1098/rspb.2024.0511

**Published:** 2024-08-07

**Authors:** Jingyao Niu, Magnus Huss, Aurélie Garnier, Anti Vasemägi, Anna Gårdmark

**Affiliations:** ^1^ Department of Aquatic Resources, Swedish University of Agricultural Sciences, Box 7018, Uppsala 75007, Sweden; ^2^ Université de Rennes, UMR 6553 CNRS ECOBIO, 263 Avenue du Général Leclerc, Rennes 35042, France; ^3^ Institute of Freshwater Research, Swedish University of Agricultural Sciences, Stångholmsvägen 2, Drottningholm 17893, Sweden; ^4^ Department of Aquaculture, Estonian University of Life Sciences, Institute of Veterinary Medicine and Animal Sciences, 46A Kreutzwaldi Street, Tartu 51006, Estonia

**Keywords:** local adaptation, climate warming, thermal evolution, trophic interaction, Eurasian perch

## Abstract

Predator responses to warming can occur via phenotypic plasticity, evolutionary adaptation or a combination of both, changing their top-down effects on prey communities. However, we lack evidence of how warming-induced evolutionary changes in predators may influence natural food webs. Here, we ask whether wild fish subject to warming across multiple generations differ in their impacts on prey communities compared with their nearby conspecifics experiencing a natural thermal regime. We carried out a common garden mesocosm experiment with larval perch (*Perca fluviatilis*), originating from a heated or reference coastal environment, feeding on zooplankton communities under a gradient of experimental temperatures. Overall, in the presence of fish of heated origin, zooplankton abundance was higher and did not change with experimental warming, whereas in the presence of fish of unheated origin, it declined with experimental temperature. Responses in zooplankton taxonomic and size composition suggest that larvae of heated origin consume more large-sized taxa as the temperature increases. Our findings show that differences between fish populations, potentially representing adaptation to their long-term thermal environments, can affect the abundance, biomass, size and species composition of their prey communities. This suggests that rapid microevolution in predators to ongoing climate warming might have indirect cross-generational ecological consequences propagating through food webs.

## 1. Introduction

Organisms respond to prevailing climate warming by means of plasticity during acclimatization and evolutionary changes over generations, or both [1]. These responses can lead to physiological and life-history trait changes in organisms [1,2]. Despite extensive efforts in investigating species [3] and community responses to warming [4,5], such studies are rarely carried out over the span of organisms’ multiple generations [2,6–9]. When studies do span multiple generations, they are rarely field-based but most often carried out under laboratory conditions that are hard to extrapolate to responses in nature [10,11], or are using space for time substitution approaches [12]. While the latter often achieve large thermal gradients, there are inevitably other biotic and abiotic differences brought by the spatial gradients that can confound the effect of differences in temperature [12]. Yet, given fast ongoing environmental changes owing to global warming, there is a pressing need to investigate the cascading effects of potential evolutionary adaptations to warming in natural predators on prey communities, to improve our ability to predict the impacts of global change on food web stability and ecosystem functioning [13].

In aquatic systems, fish commonly exert top-down control on zooplankton communities by size-specific feeding [14]. Microevolutionary adaptation or acclimatization to warming in fish physiology, life-history traits and behaviour may affect their feeding, for example via changes in their metabolism, growth, development, morphology and body size [15–18]. Metabolic changes under warming can have contrasting effects on feeding. An increased top-down effect is predicted to occur owing to an increase in metabolic rate, which is common in warmer environments [19]. This requires and also enables organisms to consume more prey or prey of larger sizes [20]. However, if they are not able to fully compensate by increasing their feeding rate, increased metabolic costs can result in lower net energetic gains in warmer environments, with less energy being allocated to activities such as growth, locomotion and even motivation to initiate predation [21,22], which could lead to a decrease in top-down effect. However, a compensatory metabolic response (and potentially adaptive) in the form of a depression in standard metabolic rate (SMR) has been found in several warm-acclimatized or warm-adapted fish species [16,23–25], probably to reduce the energy loss in a warmer environment. In such cases, individuals require less food to maintain their energy balances. Their top-down effect on prey may then be lowered. Physiological adaptation can also lead to faster development [26] or body growth [11] in warm environments. Because organisms then get a larger body size when young [15] and thus potentially a larger gape size [27], or a more robust body at a given age, this may enable them to switch their diet towards larger or better swimming prey at an earlier age compared with individuals with slower growth [11,28,29]. This might lead to differences in prey selection reflected by size- or species-dependent top-down effect or phenological change in the top-down effect and thus in its strength at a given time in the season.

Effects of adaptation or acclimatization on traits or processes that can affect feeding can play out differently depending on the extent of temperature change relative to their natal habitat temperature, and exposure time. For example, thermal tolerance or SMR often follow a hump-shaped function of the novel environmental temperature [16,30]. Indirect effects from these responses on predator top-down effects could therefore also be hump-shaped, which has been shown for attack and maximum ingestion rates [6,31–33]. Changes in top–down effects on prey communities from warming-induced responses in predators may thus not be uni-directional across temperatures.

While there is evidence for thermal adaptation in predators [16] and that thermal acclimatization in predators can shape predator–prey interactions [6,8], we still lack experimental tests of how cross-generational responses of predators to warming may influence prey community composition under different temperatures. This is an important question bridging direct microevolutionary responses in predators and ecological responses in the food web within the eco–evo feedback loop triggered by warming. Such feedbacks have been found in some aquatic predators [12], but responses in prey communities to indirect effects of multi-generational warming via trophic interactions are yet to be demonstrated in fish. Compared to no warming, the warming-affected top-down effect can lead to less or more biomass reduction, shifts in species composition and size distribution of their prey [34], as well as behavioural and morphological variations in prey [35]. Warming-induced shifts in relative size distribution or turnover rates can also result in predator–prey mismatches [28]. Food web dynamics may, therefore, change differently if prey responds to warming adaptation in predators, and may then also cascade to lower trophic levels and affect the response of ecosystem processes to warming [36,37].

Here, we investigate whether the thermal origin of larval fish affects their zooplankton prey communities under a warming gradient, through a common garden mesocosm experiment. We used fish larvae that originated from two adjacent wild populations: one with ambient temperature (hereafter unheated-origin population, UN) and the other heated for many generations (heated-origin population, H). During the experiment, fish larvae of both origins fed on one zooplankton community from the unheated area. Predators and prey were jointly exposed to a gradient of experimental temperatures, and direct measures on all trophic levels were taken. This allowed us to study the ecological consequences of warming-induced long-term responses in wild fish populations on lower trophic levels. Our findings imply that impacts of climate warming on predators across generations can have indirect, yet substantial, effects on their prey communities via shifts in trophic interactions.

## 2. Material and methods

### (a) Thermal origin of fish larvae

**Figure 1.**
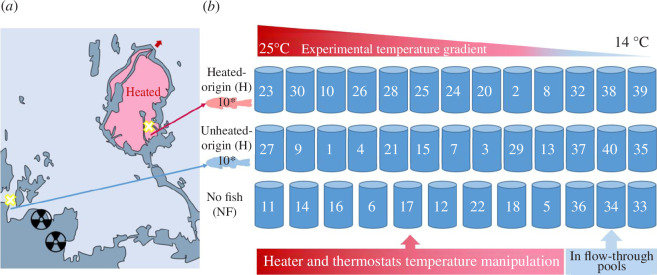
(*a*) Geographic layout of the study system: the heated area (H) and the unheated (UN) surroundings, previously separated also by a metal grid barrier at the outlet of the heated area (red arrow). The yellow crosses mark where in each area the roe strands were collected, from which larvae later were hatched to be used in the experiment. (*b*) Schematic of the experiment design testing the effect of the thermal origin of larvae (H versus UN), using 38 mesocosms (numbered 1–40, of which 19 and 31 did not exist): three rows for fish treatments (H, UN and no fish NF) and experimental temperature treatments resulting in a temperature gradient of 14–25°C achieved within each fish treatment, over the 21 day experiment period. For more details on the study system, mesocosm physical layout and temperature manipulation see electronic supplementary material, figure S1 and Experiment set-up.

The larval Eurasian perch (*Perca fluviatilis*, hereafter perch) used in the experiment originated from parental populations from two areas: (i) an area subjected to artificial heating for 41 years and (ii) an adjacent coastal area experiencing a natural temperature regime. The heated population has been residing in a 1 km^2^ heated area since 1980 and subjected to a temperature of 5–10°C above the natural, caused by heated water discharge from nearby nuclear reactors on the western Baltic Sea coast (60°43′ N 18°19′ E, figure 1*a*). The design of the set-up ensured that the two populations were exposed to similar environmental conditions, other than the heated water flow-through (for shared coastal environmental characteristics and water properties, see electronic supplementary material, table S1). A physical barrier to the exchange of fish between the heated and unheated areas was in place shortly after the enclosure construction of the heated area, resulting in a 23 year isolation of perch on there from the surrounding area. It was a 15 mm mesh size metal grid at the outlet of the heated area blocking the only narrow passage from the heated area to the surrounding coast (figure 1*a*). Plenty of studies have revealed differences in perch life-history traits and physiology in the heated area compared with the unheated area over the decades ([14,17,24,36–40]; also see electronic supplementary material, Perch larvae thermal origin: study population). Analyses using less than 10 microsatellite loci in earlier studies show significant genetic differentiation between the heated and the unheated population [38,41]. The grid was removed in 2004, which increased the probability of larger fish (>10 cm [39]) dispersing between the areas. Nevertheless, the 23 year isolation and the continuing semi-isolation of the two populations since 2004 still result in some separation between the two populations [38,41]. The combination of phenotypic trait changes and genetic shifts suggests possible warming-induced microevolution in perch from the heated area.

To study the effects on prey communities of larval perch from these two geographically close but thermally contrasting habitats, we collected 15 separate roe strands from each area on 10 May 2021, representing the H and UN perch population (electronic supplementary material, table S2). As available genetic evidence of differentiation between the two populations is limited, especially for times after the grid removal [38,41], we conducted a simple genetic analysis on 14 microsatellite loci in non-coding regions using all the 15 roe strands (as described in electronic supplementary material, Genetic analysis) to test whether these two populations are indeed separated. It showed low, but statistically significant genetic differentiation between the heated and unheated populations (electronic supplementary material, Genetic analysis). Although this is not enough to demonstrate any selection signature owing to warming or any consequent adaptation to warming, it indicates that the sampled roe strands come from two genetically differentiated populations—a precondition for our experiment.

For hatching larvae to use in the experiment, we selected eight roe strands with similar width and egg adhesion out of the 15 roe strands collected from each habitat and transferred to indoor aquaria. Each roe strand was placed in separate aquaria with approximately 100 l aerated, unfiltered coastal water with a 16 : 8 h light : dark photoperiod indoors at room temperature. The remaining seven roe strands from each population were kept in smaller aquaria (approx. 40 l) as backups (electronic supplementary material, table S2). Perch larvae started hatching from 17 May (for records of hatching status, see Record_hatchingstatus.xlsx), and on 22 May, there was an adequate amount of newly hatched larvae (<5 days old) to start the experiment naming 22 May as experiment day 0. The 10 individual perch larvae introduced in every fish-present (H and UN) mesocosm consisted of two individuals each from five selected roe strands of each of the heated and unheated origin (electronic supplementary material, table S2). These five roe strands were selected such that most larvae hatched around the same time to better control for other effects than their population of origin. At the start of the experiment, we acclimatized the fish larvae to the experimental mesocosm by gradually lowering and immersing a bag containing aquaria water and larvae into the mesocosm water.

### (b) Mesocosm experiment

We established 38 outdoor mesocosms using tanks (FlexiTank, Max Grow Shop) of a volume of approximately 400 l, diameter of 0.68 m, height of approximately 1.1 m, made of polyurethane fabric and supported by rods. Altogether, 26 of these tanks were inoculated with 10 fish larvae each (13 tanks with heated-origin and 13 with unheated-origin fish larvae) and the remaining 12 tanks were kept without fish as control (figure 1*b*). Hereafter, we refer to these as heated-origin mesocosms (H), unheated-origin mesocosms (UN) and no fish mesocosms (NF). H and UN mesocosms are jointly referred to as fish-present mesocosms. The experiment was conducted outdoors for 21 days (22 May–11 June 2021, experiment day 0–20). In these open-to-atmosphere tanks, we generated a simplified pelagic food web with plankton and fish larvae. The mesocosms were filled to have an approximately 95 cm water column with 350 l water from the unheated area (salinity approx. 5 PSU) filtered through a 50 μm mesh. One day prior to the start of the experiment (i.e. on 21 May), zooplankton communities were collected at four sites in bays and along the shore in the unheated area, partly overlapping with the site for roe strand collection in the unheated area. Plankton nets of 20 and 70 μm mesh size were lowered to the target depth (<1 m), pulled horizontally for approximately 5 min at an average speed of 1 knot (0.5 m/s), and thereafter quickly retrieved to the surface to empty the collected plankton. Active sampling was conducted for a total of 3 h. Collected plankton were kept in an approximately 700 l tank filled with filtered water from the unheated area until being added to the mesocosms. On 21 May, 7.5 l of this well-mixed plankton mixture was added to each mesocosm.

**Figure 2.**
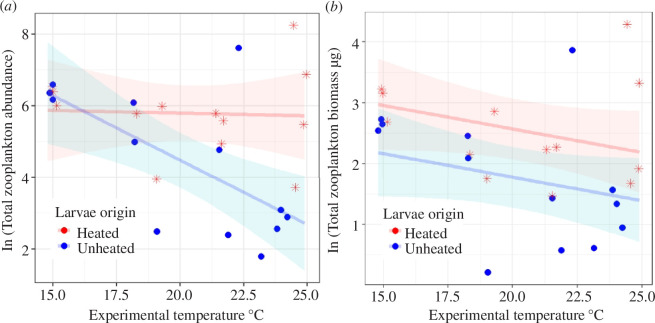
Zooplankton total abundance (*a*) and total biomass (*b*) at the end of the experiment in mesocosms with either fish larvae of heated population (H, red stars) or unheated population (UN, blue-filled points) along the experimental temperature gradient. The red and blue lines depict the predicted ln-transformed zooplankton abundance (*a*) or biomass (*b*) and the belts show their corresponding confidence intervals at 95%, using the best models (table 2, electronic supplementary material, table S7), which are ln(abundance)~origin × temperature for predicting abundance and ln(biomasss)~origin + temperature for biomass.

**Figure 3.**
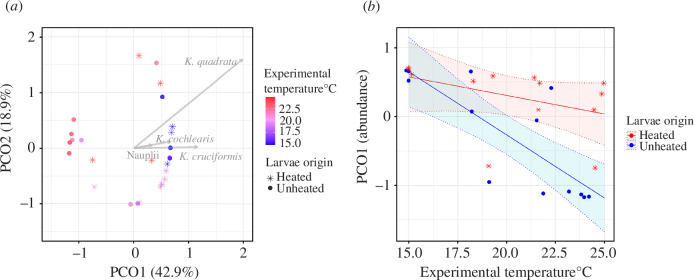
(*a*) Ordination of two main axes: PCO1 explaining 42.9% of the total variation, and PCO2, 18.9%, representing zooplankton community composition based on the abundance of different taxa in H mesocosms (star) and UN mesocosms (circles). Colour shows the mean experimental temperature in the mesocosm (from blue to red). Grey arrows show loadings of each taxonomic group on the PCO1 and PCO2 axes. The three species of *Keratella* and nauplii have the highest PCO1 scores. (*b*) Red and blue lines show that the difference in zooplankton composition (as given by PCO1) between larvae origins increased with temperature, according to PCO1 predicted by the best model in electronic supplementary material, table S7: *ln*(*PCO1~origin × temperature*). The opaque red and blue belts show the 95% confidence interval of the predicted PCO1. The red and blue points show PCO1 scores in H and UN mesocosms. For ordination and PCO1 prediction based on biomass of each taxonomic group, see electronic supplementary material, figure S6.

The experimental temperature in each mesocosm was manipulated by either heating or cooling. Heating experimental conditions were achieved using turned-on thermostats (Eheim 300 W) placed in the centre of the mesocosm, and cooling conditions were realised by surrounding the mesocosms with a flow-through system of water from the unheated area (figures 1*b* and 2). For standardization, all mesocosms had thermostats in them regardless of them being turned on or off. We deployed a temperature logger (HOBO UA-001-64 Pendant Temperature Data Logger) at 50 cm depth in each mesocosm and recorded water temperature every hour since the temperature manipulation started. After averaging temperatures over the duration of the experiment, a thermal gradient of 14–25°C was maintained among all 38 mesocosms and within each fish treatment (figure 3). On this continuous gradient, temperatures in mesocosms of each fish treatment were evenly distributed across the full gradient (figure 3). The temperature fluctuated within each mesocosm owing to exposure to natural air temperature variation (electronic supplementary material, figure 3). Each mesocosm was aerated on the bottom with approximately the same intensity of air flow using one air stone connected to an air pump (Airflow 400, IP44 230 V). The aeration saturated mesocosm water with oxygen (electronic supplementary material, Mesocosm water chemistry) and prevented temperature stratification.

### (c) Sampling and sample processing

Zooplankton and chlorophyll *a* (chl *a*) were sampled prior to the addition of fish larvae to the mesocosms (on day 0 and day −1, respectively), in the middle of the experiment (day 9) and when approaching the end of the experiment (day 19) by sampling 3.3 l water from each mesocosm at 40 cm depth using a 0.66 l Ruttner water sampler. This way, zooplankton was sampled from the water column at 40 cm depth to 16 cm without removing too much water or too many zooplankton. The water was filtered through a 70 μm mesh to keep most zooplankton on the mesh. We gently rinsed off each sample of the zooplankton collected on the mesh into a 100 ml brown bottle with tap water and added 4 ml Lugol’s iodine solution into the same bottle. To estimate chl *a*, we filtered 500 ml water through a 47 mm glass fibre filter (Whatman^TM^ GF/F) using a pressurized chamber. The GF/F filters were stored folded in aluminium foil and put in sealed bags at −20°C until they were processed for estimation of chl *a* concentration to approximate the phytoplankton biomass in our mesocosms [42]. Before adding perch larvae to the mesocosms, chl *a* and zooplankton were sampled and processed to confirm that there were no significant differences in phytoplankton and zooplankton communities among mesocosms of different treatments (electronic supplementary material, Mesocosm initial conditions). From 3 June, the remaining filtered water after sampling was returned to mesocosms to slow down the noticeable decrease in water level (mostly owing to evaporation under warm weather).

Zooplankton identification, counting and measurements were done using a stereo microscope (Leica M125C). We counted and measured individuals in 30 ml subsamples of the zooplankton mixture, and in the remaining volume, we continued counting only the taxa of which less than 50 individuals were counted in the first 30 ml. Copepoda were separated into nauplii, copepodite stages 1–3, stages 4–5 of order Calanoida (genus *Acartia* or *Eurytemora*), adults and order Cyclopoida. Cladocerans were separated into *Bosmina* sp., *Chydorus* sp. and *Podon* sp. Rotifers were identified to the genus *Keratella* (*K. cochlearis, K. quadrata* and *K. cruciformis*), *Brachionus and* class *Bdelloida*. We measured the individual body length to the nearest 0.01 mm of 20 or more individuals in each taxon for each mesocosm. Zooplankton abundance was the real count if the total volume of the sample was counted or extrapolated from a count of the first 30 ml in relationship to the sample’s total volume. Zooplankton biomass was estimated from length measurements using body length–biomass relationships (electronic supplementary material, table S3) based on the abundance. Species richness was defined as the total number of present and different taxonomic groups.

To estimate chl *a* concentrations, we processed the GF/F filters as follows: we cut each filter into half (exact proportion measured for accuracy) and put one of the halves directly into a 5 ml screw cap vial filled with 96% ethanol and then kept it in darkness at 4°C for 22.5 h for chl *a* extraction. All samples were shaken vigorously halfway through the extraction. Samples were thereafter centrifuged at 5000 r.p.m. for 5 min to sediment any particles. Three replicates of 200 µl of the supernatant of each sample were pipetted into three wells of a black solid-bottom 96-well plate. Fluorescence was measured at *λ*_ex/em_ = 444 ± 12/675 ± 50 nm using a microplate reader (Hidex Sense) and converted to chl *a* concentration (μg l^−1^) following the equation: chl *a* concentration (μg l^−1^) = (RFU − 2766.5)/194.1 (electronic supplementary material, Mesocosm initial conditions). The samples and plates were kept cool in darkness and handled as fast as possible during the process.

Perch larvae used in the mesocosms were sampled at the end of the experiment, on experiment day 20 (11 June ), by handmade drop nets of 1 mm mesh size that were slightly smaller than the mesocosm tank’s diameter. We sank the drop net vertically to the mesocosm bottom, turned it horizontally at the bottom, pulled to the surface horizontally and picked up the fish from the net using tweezers. The fish were immediately put in a benzocaine solution (200 mg l^−1^) to euthanize them and then transferred to containers with 80% ethanol for storage. If no fish were caught during five such fishing attempts, we deemed that no fish was left in the mesocosm and moved on to the next. In total, 138 fish larvae were caught. For the first three mesocosms, we repeated the same fishing process and emptied the tanks to validate the drop net method to see if there were missing fish to be caught. Despite the fact that no fish were caught during this control, we found 16 fish alive 22 days after the experiment ended (2 July) when we emptied all mesocosms. We deemed the sum of both occasions to be the best estimate of how many fish larvae survived the experiment (until 11 June). The number of fish captured on both occasions (11 June and 2 July) and the total can be found in electronic supplementary material, table S4.

Perch larvae hatched from the selected five roe strands of each origin were also sampled on 22 May after mesocosm larvae had been taken out from the hatching aquarium for inoculation, to measure their size (electronic supplementary material, tables S2, S5), as an approximation for the inoculated larvae. Thirty larvae were sampled from each aquarium using a hand net and stored in a container at −20°C with water. Body lengths of both mesocosm-caught (after experiment) and aquaria-caught (before experiment) perch larvae were thereafter measured to the nearest 0.01 mm using a stereo microscope. Fish wet weights were measured to the closest 0.1 mg after we patted them dry on both sides twice to minimize alcohol residual.

### (d) Statistical analyses

All data processing and statistical analyses were conducted in R, v. 4.0.2, R Core Team 2014 [43]. Responses in the zooplankton community at the end of the experiment were our main focus. We assembled five different response variables: zooplankton abundance, biomass, species richness, composition by taxa and composition by size. Zooplankton abundance and biomass were ln-transformed prior to analyses. The zooplankton composition by taxa was represented by scores of the first ordination axis (PCO1) of an analysis of principal coordinates made on zooplankton taxa (based on zooplankton abundance or on biomass per each taxonomic group at the end of the experiment). It was achieved in two steps: first, we calculated a distance matrix of Bray–Curtis dissimilarity indices [44] using the function vegdist in package ‘vegan’ [45] on the raw data; then we used the function capscale to conduct an analysis of principal coordinates: capscale(matrix ~ 1). We grouped calanoid copepodite stages 4–5, adult *Eurytemora* and *Acartia* and cyclopoid copepods together as Copepoda because the numbers were low (owing to fish predation, see electronic supplementary material, Fish predation). We also recorded variance found between zooplankton taxa (species scores on PCO1) to investigate whether some taxa were particularly influential in driving the variation in zooplankton composition among mesocosms. The zooplankton composition by size was estimated by the proportion of large-size (>200 μm) individuals. We found that 200 μm was a suitable threshold to separately group large- and small-sized zooplankton for their clear difference in abundance. It also separated the zooplankton species/taxonomic groups; copepods were larger than 200 μm and the three species of *Keratellas* were below 200 μm.

We used analysis of covariance (ANCOVA) to test whether the thermal origin of the larval fish (abbreviated as ‘origin’, as a categorical variable with discrete levels: H and UN), experimental temperature (as a continuous variable) and their interaction influenced these five zooplankton response variables at the end of the experiment. We then made predictions for abundance, biomass, scores of PCO1 and proportion of large individuals using the best model selected from established models using the three available variables (see table 2 for example model formulas). We identified the best model by ranking the established models based on the significance of pairwise likelihood ratio tests on sequential models of increasing complexity [46]. For zooplankton abundance, biomass and PCO1, we used linear regression in base R to establish models. For a proportion of large individuals, models were established using the R-package betareg [47].

We also tested the effect of thermal origin, experimental temperature and their interaction across the three sampling dates in the experiment on zooplankton abundance and biomass, using the model selection approach as above. The experimental temperature was calculated for each of these experiment days by averaging the hourly measurements from day 0 up until the experiment day in question. We also added experiment day to the models to account for the effect of time and the random effect of mesocosms. Models (electronic supplementary material, table S6) were established using the R-package lme4 [48].

Fish larvae survival, body length and weight at the end of the experiment were also analysed to evaluate their confounding effect on the thermal origin. Fish larvae weights were ln-transformed prior to analyses. Fish survival rate was calculated as the number of total larvae caught (electronic supplementary material, table S4) divided by 10, i.e. the initial number of fish larvae inoculated in each fish-present mesocosm. These values were then square root transformed to account for heteroscedasticity. Similarly, for zooplankton responses, we used ANCOVA to investigate whether there were fish-related variations between H and UN mesocosms.

Data visualization and processing were done using the packages within the tidyverse collection [49].

## 3. Results

Fish larvae thermal origin affected zooplankton total abundance, which also differed depending on experimental temperature (table 1, figure 2*a*). Zooplankton abundance at the end of the experiment was constant along the temperature gradient in H mesocosms, whereas it decreased with temperature in UN mesocosms (figure 2*a* and table 2). The effect of the thermal origin of the fish on zooplankton total abundance was evident throughout the experiment (electronic supplementary material, figure S4 and table S6).

**Table 1.** Results of ANCOVA on the effects of larvae thermal origin (heated/unheated), temperature and the interaction between origin and temperature on zooplankton community abundance and biomass at the end of the experiment (day 20). The asterisk symbol indicates significant results (*p* < 0.05).

zooplankton	explanatory variables	F_1,38_	*p*-value
total abundance	origin	5.84	0.024 *
	temperature	5.12	0.034 *
	origin × temperature	4.82	0.039 *
total biomass	origin	4.60	0.043 *
	temperature	2.33	0.141
	origin × temperature	1.30	0.266

**Table 2.** Result of model selection for finding the best model (marked in bold) to predict zooplankton abundance at the end of the experiment, as well as the coefficients of each included term of the best model. The established models are labelled as null, first, second and third with increasing complexity. Results of pairwise likelihood ratio tests are shown with asterisks representing the significance level. ‘Temperature’ coefficient −0.02 shows that zooplankton abundance remained nearly constant in H mesocosms and the coefficient −0.34 for the interaction 'origin × temperature' shows that zooplankton abundance decreased with temperature in UN mesocosms. Note that the coefficient for ‘origin’ 5.58 suggests that when the experimental temperature is 0°C, the abundance in UN mesocosm would be 5.58 units higher than the abundance in H mesocosms, which is of little biological meaning for our experimental temperature gradient, as the effect is taken over by the interaction term.

model selection				best model estimates	
response	model	formula	significance	terms	coefficients
ln(total zooplankton abundance + 1)	null	1			
	first	temperature	0.07	origin	5.58
	second	origin + temperature	0.02*	temperature	− 0.02
	third	origin × temperature	0.03*	origin × temperature	− 0.34

Similarly, for zooplankton total biomass, there was an effect of fish thermal origin at the end of the experiment (table 1, figure 2*b*, electronic supplementary material, table S7), but across the three sampling dates, no model was significantly better than the null model (electronic supplementary material, figure S5 and table S6). Whereas zooplankton biomass decreased with temperature in both fish treatments, at the end of the experiment it was overall higher in H than UN mesocosms over the temperature gradient (figure 2*b*, electronic supplementary material, table S7).

While species richness did not differ significantly between H and UN mesocosms (ANCOVA, *F*_1,22_ = 4.23, *p* = 0.096), the fish origin affected zooplankton compositional variation as described by PCO1 (table 3; similarly for both when based on abundance or biomass). The effect of temperature on zooplankton composition also varied with fish origin (table 3). Scores on PCO1 based on both abundance and biomass remained nearly constant with temperature in H mesocosms but decreased with temperature in UN mesocosms (figure 3*b* and electronic supplementary material, figure S6b). The four taxa that partitioned the most variance on PCO1 (shown by the four longest arrows in figure 3*a* and electronic supplementary material, figure S6a), were three species of *Keratellas* and copepod nauplii. All four taxa had positive PCO1 scores, and as PCO1 decreased with experimental temperature in UN mesocosm, this suggests that these four taxa were negatively affected by larvae originated from the unheated area and the effect accentuated with temperature (figure 3*b* and electronic supplementary material, figure S6b). In contrast, the model of the PCO1 suggests that consumption by larvae from the heated population did not alter the zooplankton composition as temperature increased (figure 3*b* and electronic supplementary material, figure S6b), although the consumption of zooplankton total biomass did increase as temperature increased (figure 2*b*).

**Table 3.** Results of ANCOVA on zooplankton composition by taxa, represented by the scores of PCO1 generated from distance matries based on abundance or biomass per each zooplankton taxonomic group. The asterisk symbols indicate significant results (*p < 0.05, **p < 0.01, ***p < 0.001). Zooplankton composition given by PCO1 was affected by the thermal origin of fish larvae (heated/unheated), experimental temperature and their interaction.

distance matrix based on	variables	*F*	*p*-value
abundance	origin	16.65	<0.001 ***
	temperature	9.85	0.005 **
	origin × temperature	5.64	0.027 *
biomass	origin	19.69	<0.001 ***
	temperature	13.4	0.001 **
	origin × temperature	7.2	0.014 *

**Figure 4.**
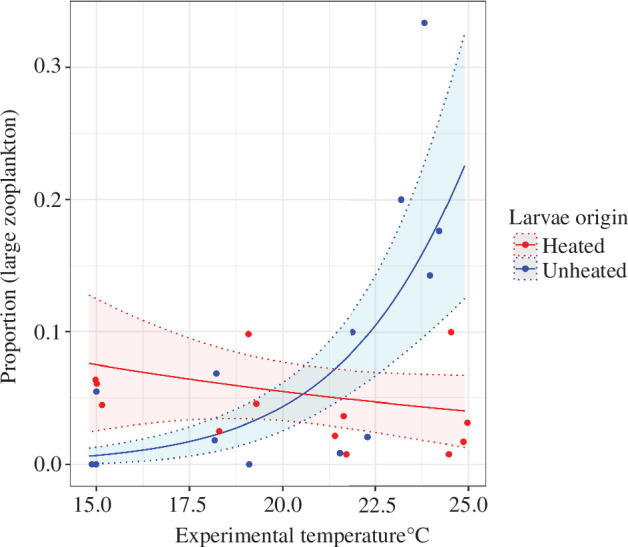
The proportion of large-sized zooplankton in H mesocosms (red points) and UN mesocosms (blue points) and predictions of it (and 95% confidence interval) made from the best model P(large zooplankton)~origin × temperature (electronic supplementary material, table S7) shown as the red and blue lines (belts for the confidence intervals). The proportion of large individuals is predicted to remain nearly constant at a low level in the H mesocosm and increase with temperature in the UN mesocosm.

Similarly, there was also an interactive effect of the thermal origin of fish and experimental temperature on the proportion of large-sized zooplankton (figure 4, electronic supplementary material, table S7). The proportion decreased with temperature in H mesocosms but increased in UN mesocosms, rendering a smaller proportion of large-sized zooplankton in mesocosms with larvae originated from the heated than unheated populations at higher temperatures, especially in the eight warmest mesocosms (figure 4). We also found a significantly smaller proportion of copepods in those H than those in UN mesocosms (electronic supplementary material, figure S7). This suggests that the larvae of heated origin affected large zooplankton more than the larvae of unheated origin. This is in line with the result found in the zooplankton taxonomic composition response, where heated-origin larvae did not affect the four small-bodied taxa as much as unheated-origin larvae did.

Fish larvae had a clear top-down effect on zooplankton, shown by lower abundance and biomass of zooplankton (electronic supplementary material, figure S8 and Fish predation), and lower species richness (ANCOVA, *F*_2,32_ = 33.94, *p* < 0.001), in fish-present mesocosms compared to NF mesocosms (note that there was no difference in the initial conditions, see electronic supplementary material, Mesocosm initial conditions). Furthermore, the effect of fish thermal origin on zooplankton responses was probably not owing to a difference in fish abundance or growth as survival and the final length of fish larvae did not differ between H and UN mesocosms (ANCOVA on survival: *F*_1,22_ = 0.44, *p* = 0.48; length: *F*_1,134_ = 2.58, *p* = 0.111). However, individual H larvae slightly gained more weight than the ones of unheated origin during the experiment (ANCOVA, *p* = 0.038, *η* ^2^ = 0.03).

Larvae originating from different thermal populations thus have a different top-down effect on the zooplankton community, indicated by the observed differences in zooplankton abundance, biomass, species composition and size composition between H and UN mesocosms. The differences found in zooplankton responses between H and UN mesocosms did not, however, cause any differences in phytoplankton biomass between H and UN mesocosms, indicated by their similar chlorophyll *a* concentrations (electronic supplementary material, figure S9, ANCOVA, *p* = 0.462). Fish larvae did, however, have indirect top-down effects on phytoplankton, as chl *a* was higher in mesocosms with fish than in NF mesocosms, both throughout and at the end of the experiment (electronic supplementary material, figure S9). Taken together, this indicates that while fish larvae had top-down effects on zooplankton that in turn affected phytoplankton, the differences in these top-down effects depending on fish thermal origin did not cascade down to phytoplankton.

## 4. Discussion

We found that the thermal origin of fish larvae affected the abundance, biomass, as well as taxonomic and size composition of their zooplankton prey community differently, depending on the temperature in a mesocosm warming experiment. While controlling for initial prey community composition and other conditions among all mesocosms, no difference in fish abundance and body length at the end was found depending on the thermal origin, suggesting that the observed effect of fish thermal origin on zooplankton was probably owing to differences in the feeding of perch larvae depending on their origin. This further suggests that perch may have responded to the long-term extensive heating in ways that resulted in a reduced top-down control on zooplankton.

The difference between zooplankton prey community responses to the presence of fish of heated and unheated origin might have arisen from a few non-exclusive mechanisms. First, perch from the chronically warmed environment may have physiologically adapted such that they require less food to sustain themselves under elevated temperatures, owing to a lowered metabolic rate [16,50]. Specifically for our study populations, adult perch from the heated area display significant metabolic thermal compensation and lowered heart rate at high temperatures, compared with adults from the unheated area [25]. Such warming-induced metabolic changes are commonly due to phenotypic plasticity [51], but evidence shows that evolutionary adaptations can also cause such metabolic changes, potentially selected to compensate for higher energy losses at high temperatures [16]. This potential lower food requirement of fish from the heated area might be why zooplankton abundance remained constant along the temperature gradient in mesocosms with larvae of heated origin, while the top-down effect of larvae of unheated origin increased with temperature, probably linked to increased feeding rates owing to increased (not-adapted) metabolism (figure 2*a*).

Second, larvae of heated origin may have reduced attack rates overall, which would directly decrease top-down effects on zooplankton. When exposed to high temperatures, some organisms display reduced attack rates even after acclimatization to warmer environments [6,50], although warming-induced evolution of fish attack rates has not yet been shown. It is also possible that perch of heated origin have adapted to feed on an already warm-adapted prey community in the heated area [17], and thus fed less efficiently when exposed to an unfamiliar prey community from the unheated area in the mesocosms, resulting in the observed lower top-down effect on zooplankton compared with the larvae of unheated origin (figure 2*b*). A follow-up experiment with a full factorial set-up manipulating the origins of both fish and zooplankton, in addition to the current experiment design, would help clarify this.

Third, larvae of heated origin may have evolved in ways that allow them to reach higher growth and development rates in high-temperature environments [26], potentially explaining the slight positive yet significant weight gain by the larvae of heated origin compared with the larvae of unheated origin. Young (though non-larval) perch of heated origin also have a faster growth rate [15] and earlier reproduction [52] in their heated home environment. Ultimately, these changes can lead to changes in body size [15,53] which determines the size of prey that larval fish are able to consume and/or prefer to feed on [54]. A larger gape size enabling an earlier shift to feed on larger-sized zooplankton [55] of larvae of heated origin could explain why we found proportionally less large-sized zooplankton and specifically fewer copepods in the presence of these larvae than of unheated-origin larvae at high temperatures (figure 4, electronic supplementary material, figure S7), and a larger negative effect of the larvae of unheated origin on the small-bodied zooplankton taxa (figure 3). This could also explain why the abundance did not decrease as much as biomass did in zooplankton communities in mesocosms with the larvae of heated origin as temperature increased (figure 2). It is also possible that the larvae of heated origin had a general shift to larger-sized zooplankton which did not change their relative prey consumption based on taxonomic groups (figure 3*b*, electronic supplementary material, figure S6b), but resulted in the increase in the total biomass with temperature (figure 2*b*).

We cannot rule out the contribution of maternal effects to the differences in fish top-down effects between the thermal origins, although we controlled for egg size of candidate roe strands (as indicated by roe strand width [55]) for the hatching of the perch larvae used in the experiment. Despite our selection effort, heated-origin roe strands were slightly wider than the unheated ones (electronic supplementary material, Roe strand collection and larvae selection). This may have resulted in the observed slightly larger body length in H larvae than in UN larvae at inoculation (electronic supplementary material, table S5), despite hatching around the same time. This difference in body length could have contributed to potential prey selection differences and thus observed differences in prey composition. However, no difference was found in the initial weight of perch larvae (electronic supplementary material, table S5), suggesting that any potential maternal effects were probably small. Nevertheless, without more comprehensive testing for the genetic base of local adaptation relating to warming and genome-wide association studies integrating physiological and life-history traits, we cannot conclude which mechanism of the four discussed above is more likely to have caused the observed prey community responses to the presence of fish of different thermal origin.

Besides the direct top-down effect of fish larvae on zooplankton, we also investigated its indirect effects on the lowest trophic level in our mesocosms. We found that the differences in top-down effect between H and UN larvae on zooplankton probably did not cascade down to affect phytoplankton community biomass as suggested by the lack of a difference in chlorophyll *a* concentration between H and UN mesocosms (electronic supplementary material, figure S9). On the contrary, the general top-down effect from larvae independent of origin cascaded down to affect phytoplankton, as evident in a difference between chl *a* concentration between mesocosms with (H and UN) and without (NF) fish (electronic supplementary material, figure S9). The lack of a fish origin effect on phytoplankton biomass might be explained by the fact that primary producers are not susceptible to the moderate changes in the feeding of top predators as the intermediate trophic levels [13], i.e. zooplankton in our experiment. It could also be that other responses in the phytoplankton communities occurred than in the variable we measured (chl *a* concentration), e.g. in phytoplankton community species or size composition.

A few factors should be taken into consideration when interpreting our results. Our mesocosms were open to the atmosphere, resulting in their exposure to one storm event and to the potential colonization of other organisms. The storm that triggered a short power shutdown reduced the experimental temperature in all mesocosms to a similar level (electronic supplementary material, figure S3). This incident did not affect the overall temperature gradient among mesocosms over the experiment period nor did it remove the variation between them. Most importantly, the storm equally affected all mesocosms regardless of their fish treatment, so our main finding of the larval thermal origin effect on zooplankton should remain valid. Furthermore, the open atmosphere conditions may be more relevant to conditions in nature and thus provide more accurate predictions than studies conducted at constant temperatures [56]. We focused on zooplankton community responses and thus did not sample non-zooplankton prey organisms, e.g. chironomids, in the mesocosms. Those other invertebrates could have been part of the larval fish diet in addition to zooplankton or they could have also been predating on zooplankton communities besides the fish larvae [57]. However, as more than 99% of the fish larvae in the mesocosms were smaller than 20 mm, they were probably unable to efficiently consume larger prey such as chironomid larvae or pupae [58]. We found no difference in the presence of chironomids between H and UN mesocosms (electronic supplementary material, Other organisms). Thus, we believe the observed differences in zooplankton prey communities are driven by the thermal origin of fish larvae. Other factors that potentially confound the heated and unheated origin of our experimental larvae are environmental factors other than temperature that vary between the areas from which H and UN larvae originated. However, as the two areas were once a single area, environmental differences probably stem from the four-decade-long heating. We also expect any such variation (e.g. in primary production, vegetation biomass) to be outweighed by the unusually large temperature difference for natural systems (i.e. 5–10°C) between areas in terms of its effect on the ecosystem and the organisms therein. Therefore, the observed effects of larval origin on their zooplankton prey are most probably a consequence of long-term warming. Moreover, the long-term warming has occurred at the scale of a whole ecosystem, which makes findings in our specific study populations highly relevant in the context of global warming.

In conclusion, we show from direct measurements of zooplankton prey communities that wild predators’ responses to multi-generational whole-ecosystem warming can induce variation in their top-down effects. The overall reduced feeding found in the larvae of heated origin was specifically evident in high-temperature environments, indicating that warming-induced changes in top-down effects may buffer potential negative effects of warming on the prey communities. Our experimental findings using a perch population heated for multiple generations aids the understanding of how warming may affect eco–evo feedback loops by showing that potential adaptations in predators to long-term warming can propagate to affect their prey community via feeding interactions. We, therefore, call for experiments generalizing our novel findings, to test whether responses to multi-generational warming in other fish and/or older life stages would cascade down to affect lower trophic levels and to further investigate the mechanisms by incorporating evolutionary and quantitative genetic methods—to infer whether these responses to warming are owing to local adaptation, maternal effects or others, in order to better predict community-wide impacts.

## Data Availability

The data used in this manuscript can be accessed from the electronic supplementary material and Zenodo [59]. Supplementary material is available online [60].
